# Obesity is associated with greater cognitive function in patients with type 2 diabetes mellitus

**DOI:** 10.3389/fendo.2022.953826

**Published:** 2022-10-24

**Authors:** Zhenhua Xing, Chen Long, Xinqun Hu, Xiangping Chai

**Affiliations:** ^1^ Department of Emergency Medicine, Second Xiangya Hospital, Central South University, Changsha, China; ^2^ Emergency Medicine and Difficult Diseases Institute, Central South University, Changsha, China; ^3^ Department of General Surgery, The Second Xiangya Hospital, Central South University, Changsha, Hunan, China; ^4^ Department of Cardiovascular Medicine, The Second Xiangya Hospital, Central South University, Changsha, Hunan, China

**Keywords:** body mass index, waist circumference, cognitive function, obesity, type 2 diabetes mellutus

## Abstract

**Background:**

The impact of obesity on cognitive function in patients with type 2 diabetes mellitus (T2DM) remains controversial. This study aimed to evaluate whether obesity, assessed by body mass index (BMI) was associated with cognitive function among T2DM patients and whether the effect of obesity on cognitive function was through brain structure.

**Methods:**

This was a *post-hoc* analysis of the Action to Control Cardiovascular Risk in Diabetes–Memory in Diabetes (ACCORD-MIND) study. The cognitive test battery included the Digit Symbol Substitution Test (DSST), Mini-Mental State Exam (MMSE), Rey Auditory Verbal Learning Test (RAVLT), and STROOP test, which were administered at baseline, and at 20, 40, and 80 months. A subgroup (n = 614) of the ACCORD-MIND study underwent MRI scanning at baseline and at 40 and 80 months. The total brain volume (TBV), abnormal white matter volume (AWM), abnormal gray matter volume (AGM), and abnormal basal ganglia volume (ABG) were estimated. The outcomes of this study were cognitive function and brain structure.

**Results:**

In the adjusted analyses, BMI was positively associated with the MMSE (β:0.08, 95%CI,0.01-0.16, per standard deviation [SD] increase) and RAVLT scores (β:0.09, 95%CI,0.01-0.18). It was also associated with a greater TBV (β:7.48, 95%CI,0.29-14.67). BMI was not associated with the DSST or STROOP scores, and AWM, AGM, ABG. Mediation analysis found that the effect of BMI on MMSE/RAVLT was mediated through TBV.

**Conclusion:**

Obesity may be associated with greater cognitive function and the effect of BMI on cognitive function may be mediated by TBV among patients with T2DM.

**Clinical Trial Registration:**

http://www.clinicaltrials.gov, identifier NCT00000620.

## Introduction

Obesity is associated with an increased risk of cardiovascular disease (CVD) and cancer, which places it among the top public health challenges (1–3). The prevalence of obesity is high and continues to rapidly increase worldwide (4). Meanwhile, the prevalence of cognitive impairment is increasing as the population ages. Recent studies found that several types of pathological changes and metabolic diseases associated with obesity were also associated with cognitive impairment or dementia (5, 6). Obesity can also lead to high rate of diabetes and hypertension, which further accelerates arterial aging, indexed as arterial stiffness, which in turn leads to higher blood pressure; these factors have been recognized independent risk factors for cognitive damage (6–8). Despite this evidence, the association between obesity and cognitive impairment remains controversial (9–13). This is possibly due to the heterogeneity of the studied populations (14). Midlife obesity may be a risk factor for cognitive impairment, but not for those in special groups, such as type 2 diabetes mellitus (T2DM) patients and the elderly population (15, 16).

T2DM is a risk factor for cognitive decline (17) and dementia. Patients with T2DM are more likely to be overweight and obese than those without T2DM (18). Nevertheless, only a few studies have examined the relationship between obesity and cognitive function in the context of T2DM, showing that obesity is a risk factor for cognitive impairment even in the elderly (19, 20). Moreover, different cognitive tests lack consistency, and their results may be affected by different cognitive tests. Thus, multiple cognitive tests are needed (21).

Most previous studies focusing on the relationship between obesity and cognitive function lacked brain imaging evidence, thereby limiting their conclusions (9, 10). Meanwhile, previous studies found that obesity was associated with a smaller regional brain volume and higher coherence but a lower magnitude of white matter microstructure (22, 23). However, the extent to which these structural brain changes affected cognitive function remained unclear. Studies that combined cognitive function and structural brain assessments might provide information on the relationship between obesity and cognitive function. By simply acting prevention, particularly on body weight reduction, it could be possible to reduce the incidence of cognitive decline (24). Thus, this study aimed to evaluate the association between obesity and cognitive function as well as the association between obesity and brain imaging findings. Moreover, this study aimed to analyze the correlation between structural brain imaging findings and cognitive function in T2DM patients and whether the effect of obesity on cognitive function was through brain structure.

## Methods

The Action to Control Cardiovascular Risk in Diabetes (ACCORD) study was a randomized, double two-by-two factorial parallel treatment trial that aimed to determine whether intensified, compared with standard management of blood glucose (glycosylated hemoglobin [HbA1c] of <6.0% vs. that in the range of 7.0–7.9%), blood pressure (systolic blood pressure [SBP] of <120 vs. <140 mmHg), or lipid levels (placebo vs. fenofibrate), resulted in the improvement of cardiovascular outcomes in patients with T2DM (3, 18). The participants with T2DM were aged 45-79 years and were recruited across 77 clinics in North America. They had high HbA1c levels (>7.5%) and a high risk of CVD events (at least two additional risk factors for cardiovascular disease [dyslipidemia, hypertension, current status as a smoker, or obesity]).

**Figure 1:**
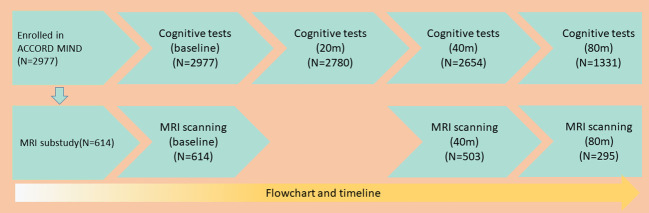
The study’s flowchart and timeline.

The ACCORD–Memory in Diabetes (ACCORD-MIND) sub-study included 2,977 participants recruited at 52 of 77 clinics in North America to test whether these interventions could reduce the rate of cognitive decline and magnetic resonance imaging (MRI)-detected structural brain changes. Within the ACCORD-MIND sub-study, a subset of participants (n = 614) underwent MRI scanning. The intensified control of blood glucose levels was halted on February 6, 2008 due to its associated increase in mortality rates. Participants in the intensive blood glucose control group were then moved to the standard group. The ACCORD-MIND study follow-up assessments continued, providing four measures of cognition and three measures of brain structure throughout the study period. As shown in **Figure 1**, 2,780 participants completed the tests at least two times; 2,654 participants completed the tests three times; and 1,331 participants completed the tests four times. A total of 503 participants underwent MRI at least twice, while 295 participants underwent MRI thrice.

### Exposure and covariates

Baseline and annual follow-up data on the participants’ demographic and clinical characteristics, including any treatments, were collected through physical examination, interviews, and laboratory tests. The parameters that we aim to assess were body mass index (BMI) and waist circumference (WC). BMI was defined as weight in kilograms divided by the square of the height in meters. Participants were classified according to BMI standards: normal, 18.5–24.9; overweight, 25–30; class I obesity, 30.1–35; class II obesity, 35.1–39.9; and class III obesity, BMI ≥40. The normal weight group was used as a reference. WC was divided into sex-specific quintiles, and the first quintile was used as a reference. BMI was used as the main indicator of obesity in our study, while WC was used in sensitivity analysis. Since there were only small fluctuations in BMI and WC as well as desynchronized measurement of BMI or WC and cognitive function (every 1 year versus every 20 months) during the follow-up period, we used the baseline BMI or WC values as exposure variables in the present study (25).

We considered covariates and confounders previously associated with obesity, cognitive function, or brain structure changes. These included age, race, sex, education level, follow-up period, glycemic control strategy, CVD history, heart failure, depression, smoking status, HbA1c level, estimated glomerular filtration rate (eGFR), high-density (HDL) and low-density lipoprotein (LDL) levels, systolic blood pressure (SBP), and diastolic blood pressure (DBP) (26–28). Smoking status was categorized as “never/former smoker” versus “current smoker” (last 30 days). Education levels were categorized as below high school, high school graduate, some college years, and college graduate or higher.

### Cognitive tests

The cognitive test battery included the Digit Symbol Substitution Test (DSST), Mini-Mental State Examination (MMSE), Rey Auditory Verbal Learning Test (RAVLT), and STROOP test. The cognitive outcomes were the DSST, MMSE, RAVLT, STROOP test results.

The DSST is a subset of the Wechsler Adult Intelligence Scale (third edition) that assesses a wide variety of cognitive domains, including visual motor speed, learning capacity, sustained attention, and working memory. The DSST is easy to administer and is not language-specific. It has been used extensively to measure cognitive function and predict future cognitive decline in patients with or without T2DM (29). The DSST has a wide distribution of scores in patients with T2DM. It is a test of psychomotor function and speed and is believed to be a good indicator of vascular cognitive impairment (30). Thus, the outcome score reflects the number of correct entries.

The MMSE, with scores ranging from 0 (worst) to 30 (best) points, is useful for cognitive impairment screening, especially among older adults. In particular, it identifies cases that may require further evaluation (31, 32).

The RAVLT, wherein a higher score indicates better neurologic function, is widely used in epidemiological studies to assess verbal memory by evaluating word registration and recall (31, 32). The outcome score represents the total number of words recalled.

The STROOP test evaluates executive function, which is mainly attributable to the frontal lobe. It reflects the ability to view complex visual stimuli, direct attention, and respond to one attribute, while inhibiting the response to another (32). The outcome score reflects the time required to complete the test, with higher scores indicating worse executive function. Therefore, the STROOP score increases with time (20,40, and 80 months) compared with the baseline.

### MRI scanning

MRI scanning protocols, quality-control procedures, and subsequent image analysis programs were previously described in detail (32). The MRI scanning protocol was used to acquire region-specific imaging data to identify brain areas that were vulnerable to T2DM or those with white matter lesion load and infarct lesions. The brain MRI outcomes were total brain volume (TBV), abnormal white matter volume (AWM), abnormal gray matter volume (AGM), abnormal basal ganglia volume (ABG), and their progression during the follow-up period. The purpose of brain MRI outcome assessment was to investigate the potential mechanism associated with obesity, which might have affected cognitive function.

The TBV may be used to indicate mixed vascular and neurodegenerative brain lesions and cerebral atrophy, which can severely affect cognitive function (33–35). AWM may reflect diffuse and focal ischemia, demyelination, and inflammatory processes associated with severely impaired cognitive function (36, 37). Recent studies showed that brain areas with AGM volumes might be associated with cognitive impairment. Participants with global loss or regional alterations in gray matter volume were at greater risk of future cognitive impairment (38, 39). The basal ganglia are of great interest, given the high concentration of dopaminergic neurons and receptors in these structures and their crucial role in cognition, including attention, working memory, and goal-directed behavior (40, 41).

### Statistical analyses

All statistical analyses were performed using the Stata/SE Version 15.1 (StataCorp LP, College Station, TX, USA), and all statistical tests were two-tailed, with a 5% level of statistical significance. Baseline participant characteristics were reported as means (standard deviation, SD) and percentages, which were compared using one-way analysis of variance and the chi-square or Fisher exact tests, as deemed appropriate. Generalized estimating equations (GEE) with an exchangeable correlation structure were used to assess the relationship between BMI with cognitive function or MRI findings. We used three models in total, including model 1, unadjusted; model 2, adjusted for age, race, sex, follow-up period, and education level; and model 3, adjusted for age, race, sex, follow-up period, education level, CVD history, heart failure, depression, smoking status, HbA1c level, eGFR, HDL, LDL, SBP, and DBP. WC was used as a continuous variable in sensitivity analysis to test the robustness of the relationship between obesity and predefined outcomes. Since medications might affect weight, we further adjusted medications in addition to model 3 in the sensitivity analysis. Subgroup and interactive analyses were performed by age (age ≥ 75 years or <75 years), sex, race, and current smoking status. We also compared the baseline characteristics of patients with cognitive function and MRI data at 80 months with those patients without data at 80months to identify potential selection bias caused by loss to follow-up (42). The MRI outcomes and cognitive function tests were treated as time-varying variables measured at baseline, 40 weeks, and 80 weeks. We further evaluated the potential mediating effect of MRI results on the observed relationship between BMI and cognitive outcomes (43).

## Results

Of the 2,977 ACCORD-MIND participants, 1,331 (45%) completed the 80th month visit. Of the 614 MRI sub-study participants, 295 (48%) underwent an MRI scan at 80 months (**Figure 1**). The baseline characteristics of the participants are shown in **Table 1**. Except for the STROOP score, the DSST, MMSE, and RAVLT scores were increased significantly with an increasing BMI. Obese participants were more likely to be young, female, white, diagnosed with depression, and with low HDL levels. The educational background differed among BMI groups. Participants with a normal weight had higher rate of college education than those in the other BMI groups. The correlation between BMI and WC was 0.79 (P < 0.01).

**Table 1:** Baseline demographic, clinical, and laboratory examination characteristics by BMI category.

	Normal	Overweight	Class I obesity	Class II obesity	Class III obesity	P-value
**N**	165	779	1011	675	347	
**BMI (kg/m^2^, SD)**	23.48 ± 1.35	27.83 ± 1.43	32.47 ± 1.47	37.18 ± 1.52	42.31 ± 1.56	<0.01
**WC (cm, SD)**	87.96 ± 7.48	97.52 ± 7.92	107.19 ± 8.37	116 ± 9.58	125.70 ± 9.85	<0.01
**DSST (, SD)**	49.04 ± 17.10	50.53 ± 16.25	53.02 ± 15.61	53.96 ± 15.33	54.62 ± 15.75	<0.01
**MMSE (, SD)**	27.00 ± 2.69	27.07 ± 2.73	27.41± 2.59	27.70 ± 2.27	27.78 ± 2.29	<0.01
**RAVLT (, SD)**	7.13 ± 2.70	7.10 ± 2.46	7.50 ± 2.53	7.74 ± 2.55	8.21 ± 2.54	<0.01
**STROOP (, SD)**	32.67 ± 17.82	33.41 ± 18.49	31.37 ± 15.26	30.80 ± 15.66	32.72 ± 17.51	0.02
**Age (years, SD)**	64.44 ± 6.34	64.17 ± 6.16	63.25 ± 5.91	61.88 ± 5.12	61.36 ± 5.02	<0.01
**Male (n, %)**	104 (63.03%)	467 (59.95%)	547 (54.10%)	336 (49.78%)	135 (38.90%)	<0.01
**RACE (n, %)**						<0.01
**Non-White**	91 (55.15%)	295 (37.87%)	271 (26.81%)	160 (23.70%)	86 (24.78%)	
**White**	74 (44.85%)	484 (62.13%)	740 (73.19%)	515 (76.30%)	261 (75.22%)	
**Glycemic control strategy (n, %)**						0.94
**Standard**	80 (48.48%)	396 (50.83%)	521 (51.53%)	337 (49.93%)	174 (50.14%)	
**Intensive**	85 (51.52%)	383 (49.17%)	490 (48.47%)	338 (50.07%)	173 (49.86%)	
**CVD history (n, %)**	37 (22.42%)	244 (31.32%)	298 (29.48%)	200 (29.63%)	90 (25.94%)	0.12
**Education (n, %)**						<0.01
**Less than high school**	24 (14.55%)	119 (15.28%)	128 (12.66%)	80 (11.85%)	41 (11.82%)	
**High school graduate**	40 (24.24%)	191 (24.52%)	269 (26.61%)	177 (26.22%)	92 (26.51%)	
**Some college**	36 (21.82%)	251 (32.22%)	355 (35.11%)	248 (36.74%)	137 (39.48%)	
**College graduate or more**	65 (39.39%)	218 (27.98%)	259 (25.62%)	170 (25.19%)	77 (22.19%)	
**Depression**	33 (20.00%)	172 (22.08%)	251 (24.83%)	208 (30.81%)	117 (33.72%)	<0.01
**Smoker (n, %)**	65 (46.10%)	332 (48.75%)	458 (50.78%)	315 (51.22%)	153 (48.26%)	0.70
**eGFR (ml/min/1.73, SD)**	90.16 ± 22.35	89.90 ± 23.32	90.08 ± 22.70	90.44 ± 24.65	89.58 ± 24.49	0.99
**LDL (mg/dL, SD)**	107.33 ± 35.04	102.14 ± 32.72	102.98 ± 33.91	103.01 ± 31.87	106.78 ± 36.56	0.16
**HDL (mg/dL, SD)**	48.17 ± 15.18	43.29 ± 11.64	41.41 ± 10.64	42.29 ± 10.84	42.15 ± 11.46	<0.01
**SBP (mmHg, SD)**	135.01 ± 19.62	135.72 ± 17.30	135.69 ± 17.89	135.18 ± 17.20	134.40 ± 17.48	0.79
**DBP (mmHg, SD)**	72.81 ± 10.97	73.25 ± 10.27	75.30 ± 10.71	75.45 ± 10.10	76.43 ± 11.20	<0.01
**Medication**						
**Metformin**	107 (64.85%)	493 (63.29%)	681 (67.36%)	431 (63.85%)	228 (65.71%)	0.42
**Thiazolidinedione**	31 (18.79%)	166 (21.31%)	229 (22.65%)	193 (28.59%)	95 (27.38%)	<0.01
**Insulin**	5 (3.03%)	75 (9.63%)	98 (9.69%)	86 (12.74%)	47 (13.54%)	<0.01
**Statin**	102 (63.35%)	529 (68.08%)	677 (67.30%)	427 (63.45%)	208 (60.12%)	0.05
**Sulphonylurea**	8 (4.85%)	15 (1.93%)	20 (1.98%)	10 (1.48%)	6 (1.73%)	0.10

BMI, Body mass index; DSST, Digit Symbol Substitution Test; MMSE, Mini-Mental State Exam; RAVLT, the Rey Auditory Verbal Learning Test; CVD, Cardiovascular disease; HbA1C, Glycosylated hemoglobin; eGFR, Estimated glomerular filtration rate; HDL, high density lipoprotein; LDL, Low density lipoprotein; SBP, Systolic blood pressure; DBP, diastolic blood pressure.

Patients with normal weight had the lowest DSST scores at both baseline and throughout the follow-up period. This was followed by patients who were overweight, then patients with class III obesity, who had the highest DSST scores (P for trend < 0.01, **Supplementary Figure 1**). An increase in BMI of 1 SD (5.32kg/m^2^) corresponded to a DSST score increase of 1.82 points (95% confidence interval [CI]: 1.28–2.35, P < 0.01) (**Table 2**). However, this association was not observed in adjusted models 2 and 3 (coefficient of each 1 SD increase in BMI: -0.01; 95% CI: -0.48–0.46; model 3). In the same analyses, using WC instead of BMI, there was no association between WC and the DSST scores (model 3; **Supplementary Table 1**). Similarly, neither a higher BMI nor WC was associated with a lower STROOP score (model 3, **Table 2**, **Supplementary Table 1**). However, we found that in the fully adjusted models (model 3), every one-SD increase in BMI was associated with an increase in the MMSE score of 0.08 (95% CI: 0.01-0.16) and an increase in the RAVLT score of 0.09 (95% CI: 0.01-0.18), although the effect was small (**Table 2**). In the same analyses using WC instead of BMI, there was a positive association between WC and the MMSE scores (coefficient of each 1 SD increase of WC: 0.15; 95% CI: 0.07–0.22; model 3; **Supplementary Table 1**), whereas there was no association between WC and the RAVLT scores (**Supplementary Table 1**).

**Table 2:** Association between BMI and cognitive test results at baseline and during the follow-up period, adjusted for potential confounders.

	Model 1	Model 2	Model 3
**DSST (per SD increase)**	1.82(1.28, 2.35)*	-0.06(-0.51,0.38)	-0.01(-0.48,0.46)
**MMSE (per SD increase)**	0.24(0.16, 0.32)*	0.08(0.00, 0.16)*	0.08(0.01, 0.16)*
**RAVLT (per SD increase)**	0.36(0.27, 0.45)*	0.07(-0.01, 0.15)	0.09(0.01, 0.18)*
**STROOP (per SD increase)**	-0.58(-1.08, -0.07)*	0.66(0.18, 1.14)*	0.50(-0.01, 1.00)

Model 1: unadjusted.

Model 2: adjusted for age, sex, race, glycemic control strategy, education levels, and follow-up period.

Model 3: adjusted for age, sex, race, glycemic control strategy, education levels, and follow-up period, CVD history, heart failure, depression, current smoker, glycosylated hemoglobin, estimated glomerular filtration rate, high density lipoprotein, Low density lipoprotein, systolic blood pressure, diastolic blood pressure.

DSST, Digit Symbol Substitution Test; MMSE, Mini-Mental State Exam; RAVLT, the Rey Auditory Verbal Learning Test;

*P < 0.05.

Participants with a normal weight had the lowest TBV during the follow-up period, which were then followed by participants with class III obesity (**Supplementary Figure 2**). In the adjusted model 3, an increased BMI was associated with a higher TBV. An increase of 1 SD in BMI resulted in a TBV increase of 7.48 cm^3^ (95% CI: 0.29–14.67, **Table 3**). This association was verified through sensitivity analysis, where each 1 SD increase of WC resulted in a TBV increase of 9.09 cm^3^ (95% CI: 2.14–16.05, **Supplementary Table 2**). Participants with normal weight had the highest AWM and ABG volumes, which were followed by patients who were overweight and those with class I–III obesity. Participants with class III obesity had the lowest AGM volumes throughout the entire follow-up period (**Supplementary Figure 2**). However, neither BMI nor WC showed a robust association with AMW, AGW, nor ABG volumes after adjusting for potential confounders (**Table 3**, **Supplementary Table 2**).

**Table 3:** Association between BMI and brain structure area at baseline and during the follow-up period, adjusted for potential confounders.

	Model 1	Model 2	Model 3
**TBV (per SD increase)**	1.09 (-6.39,8.59)	3.11 (-3.42, 9.98)	7.48 (0.29, 14.67)*
**AWM (per SD increase)**	-0.66 (-1.04, -0.28)	-0.39 (-0.78, 0.00)	-0.23 (-0.64, 0.17)
**AGM (per SD increase)**	-0.04 (-0.10, 0.02)	-0.02 (-0.09,0.04)	0.00 (-0.07, 0.07)
**ABG (per SD increase)**	-0.07 (-0.12, -0.02)	-0.05 (-0.10, 0.00)	-0.04 (-0.10,0.01)

Model 1: unadjusted.

Model 2: adjusted for age, sex, race, glycemic control strategy, education levels, and follow-up period.

Model 3: adjusted for age, sex, race, glycemic control strategy, education levels, and follow-up period, CVD history, heart failure, depression, current smoker, glycosylated hemoglobin, estimated glomerular filtration rate, high density lipoprotein, Low density lipoprotein, systolic blood pressure, diastolic blood pressure.

TBV: Total brain volume; AWM: Abnormal white matter; AGM: Abnormal grey matter; ABG: Abnormal basal ganglia.

*P < 0.05.

Robust results were also obtained upon adjusting for medication intake (**Supplementary Table 3**). **Supplementary Figures 3, 4** present the association between BMI and predefined outcomes in the different subgroups. Race, sex, glucose control strategies, and smoking statues did not interact the association between BMI and our predefined outcomes. Age interacted the association between BMI and TBV, older obese patients had higher TBV compared with their counterpart with younger age. Furthermore, we did not find baseline characteristics differ between patients with data at 80 months and those without which indicated loss to follow-up is random.

A higher TBV was associated with a higher cognitive function measured by DSST, MMSE, RAVLT, and STROOP; however, AWM showed a negative association. A higher ABG was associated with a poor cognitive function measured by DSST, RAVLT, and STROOP except for MMSE, whereas AGM was only negatively associated with DSST (**Supplementary Table 4**). A higher BMI was associated with TBV, and MMSE/RAVLT; and higher TBV was associated with higher MMSE/RAVLT. The relationship between BMI and MMSE/RAVLT was attenuated and became none statistical significance (coefficient of each 1 SD increase in BMI: 0.008, 95% CI: -0.16–0.15 for MMSE; 0.08,95%CI: -0.10-0.27 for RAVLT) after the addition of TBV in the model 3. Therefore, the effect of BMI on MMSE/RAVLT was mediated through TBV.

## Discussion

In the *post-hoc* analysis of the ACCORD-MIND study, we found that a higher BMI was associated with a better cognitive function, as evaluated by the MMSE and RAVLT scores and as evidenced by a greater TBV. Furthermore, the effect of BMI on MMSE/RAVLT was mediated through TBV.

Previous studies found that obesity in middle-aged individuals was associated with an increased risk of cognitive impairment or dementia (44–46). Obesity may lead to several metabolic changes, such as hypertension. Hypertension accelerates arterial aging, which may impair cognitive function (7). While this association in the elderly was more complex, study findings were inconsistent (6, 47). Several mechanisms may explain this controversy. One of which are the results of the cognitive test (21, 48) and the indicators of obesity, including BMI and WC (49), which have lacked consistency. We found that BMI was positively associated with the MMSE and RAVLT scores rather than the DSST or STROOP scores. However, WC was only positively associated with the MMSE scores. Solely using the DSST or STROOP test results to assess cognitive function may not provide a clear association between obesity and cognitive function. Individual cognitive tests or obesity indicators may not sufficiently evaluate the overall cognitive function or effects of obesity. Multiple cognitive tests or obesity indicators are required to fully assess the association between cognitive function and obesity. Furthermore, cognitive test results are inevitably affected by social and psychological factors, such as educational attainment and race. The present study found that obesity was positively associated with cognitive function measured by the DSST; however, this association was not observed after adjusting for the aforementioned social and psychological factors. Moreover, our previous studies found a protective effect of BMI among participants who were overweight or had mild obesity. In particular, these participants were less susceptible to premature mortality (42). In addition, elderly patients were more likely than their middle-aged counterparts to lose weight, and weight loss was positively associated with all-cause mortality and dementia (25, 50). In fact, Zhai et al. found that weight loss might predate the onset of dementia by as much as 10 years (51).

Although obesity may protect against cognitive function decline and brain structural changes, it also impairs lower baseline prefrontal metabolism, which may cause impaired performance reported in obese individuals on some cognitive tests of executive function (52). Therefore, obese individuals did not have better executive function measured by DSST or STROOP, though they had better memory.

The MRI findings observed during the follow-up period supported the association between obesity and cognitive function. Mediation analysis showed that TBV mediated the association between BMI and cognitive function. Obese patients tend to have greater TBV and lower TBV decline, which might account for better cognitive function. However, previous studies found an association between obesity and a lower TBV as well as a higher AWM volume in healthy middle-aged adults (53, 54). This association in older adults or T2DM patients was more complex, and previous study findings were inconsistent (54, 55). Such discrepancies might be due to the population heterogeneity among studies. Studies conducted in middle-aged individuals showed a positive relationship between obesity and increased all-cause mortality and dementia risk. Meanwhile, findings from studies involving T2DM or elder patients were conflicting. The harmfulness of obesity may be attenuated in the older population, and mild obesity may be a predictor of decreased mortality or high TBV (42, 54). In addition, obesity may attenuate concomitant TBV decline, while its protective role in elderly population has been shown in other studies (23, 54, 56). These findings suggest that obesity may help preserve brain volume in older adults. Consistent with these findings, the present study suggests that obesity may be protective against cognitive function decline and brain structure changes captured by MRI among T2DM participants with a high CVD risk. Further, inconsistent image analysis and subsequent predefined MRI outcomes among studies may partly account for these discrepancies. Although West et al. found that a greater BMI was associated with smaller volumes of the inferior frontal and middle temporal gyri, the difference among BMI groups was rather small (55). The extent to which these small changes affected cognitive function remained unclear, thereby raising questions regarding the clinical significance of small changes in imaging markers. For validation purposes, imaging changes suggesting a disease-modifying effect have to predict clinical outcomes; however, West et al. did not indicate whether these small changes were enough to affect cognition (55). Our present study demonstrated that a higher BMI was associated with larger TBV, whereas a larger TBV was associated with better cognitive function. TBV is sensitive and is a powerful marker of disease progression, wherein a smaller value could predict future cognitive disorders (18). This may explain the association between obesity and lower TBV decline rather than other imaging outcomes among patients with T2DM.

### Study strengths and limitations

The ACCORD-MIND trial approximately repeated assessments for four times with multiple cognitive tests over a long follow-up period, helping to reduce both random and non-random errors of the presented estimates. The large sample size and multiple assessments are the strengths of this study. Our study used BMI and WC as measures of obesity. The consistent results of analyses using different indicators of obesity and the combination of cognitive tests and brain MRI imaging suggest the robustness of our results. We further found that TBV volume was positively associated with cognitive function. Our present study combined cognitive function and structural brain assessments together, which corroborated each other.

However, the present study had several limitations. Firstly, BMI and WC were measured at baseline and were not updated over time due to desynchronized measurements of BMI or WC and cognitive function (1 year versus 20 months) during the follow-up period. Second, participants were from the United States and Canada and at a high risk of CVD; Thus, the present findings might not be applicable to other populations. Moreover, our study did not evaluate and adjust for nutritional status, exercise habits, ventricular mass, and arterial stiffness, which may affect cognition and MRI findings. Fourthly, BMI is an imperfect indicator of obesity, as it does not differentiate between lean body mass and fat mass, thereby limiting its ability to reveal the true health effects. However, we also use WC as a measure for obesity in the sensitivity analysis. The associations between obesity and cognition or MRI results were also robust. We and others have recently demonstrated that WC is a comparable measurement of obesity compared with fat mass (57, 58). Fifthly, we should notice that all tests of hypotheses use P < 0.05 as the significance criterion, with no adjustment for the numerous tests. The beta value was relatively small (0.08 per SD increase in BMI for MMSE, 0.09 for RAVLT), the real value of our present study might be not that obesity was associated with higher cognitive function, but obesity did not impair cognitive function in T2DM patients.

## Conclusions

Obesity may be associated with greater cognitive function and the effect of BMI on cognitive function may be mediated by TBV among patients with T2DM.

## Data availability statement

The original contributions presented in the study are included in the article/**Supplementary Material**. Further inquiries can be directed to the corresponding author.

## Ethics statement

The studies involving human participants were reviewed and approved by accord ethics committee. The patients/participants provided their written informed consent to participate in this study.

## Author contributions

ZX and XC designed the study and provided methodological expertise. ZX, CL and XH drafted the manuscript. CL revised the manuscript. All authors have read, provided critical feedback on, and approved the final manuscript.

## Funding

This work was supported in part by National Natural Science Foundation of China 82000298 and Natural Science Foundation of Hunan Province 2021JJ40883 to ZX. This work was also partly supported by Natural Science Foundation of Hunan Province 2021JJ40850 to CL.

## Conflict of interest

The authors declare that the research was conducted in the absence of any commercial or financial relationships that could be construed as a potential conflict of interest.

## Publisher’s note

All claims expressed in this article are solely those of the authors and do not necessarily represent those of their affiliated organizations, or those of the publisher, the editors and the reviewers. Any product that may be evaluated in this article, or claim that may be made by its manufacturer, is not guaranteed or endorsed by the publisher.
