# Supplementary material for: Obesity is associated with greater cognitive function in patients with type 2 diabetes mellitus
Source: Front Endocrinol (Lausanne). 2022 Oct 24;13:953826. doi: 10.3389/fendo.2022.953826 (PMC9637978; doi:10.3389/fendo.2022.953826)

**Obesity is associated with** **greater cognitive function in patients with type 2 diabetes mellitus**

Zhenhua Xing, MD^1,2^; Chen Long, MD^3^; Xinqun Hu, MD^4^*; Xiangping Chai, MD^1,2^*

1 Department of Emergency Medicine, Second Xiangya Hospital, Central South University, Changsha, 410011, China.

2 Emergency Medicine and Difficult Diseases Institute, Central South University, Changsha, 410011, China.

3 Department of general surgery, The Second Xiangya Hospital, Central South University, Changsha，Hunan 410011，China

4 Department of Cardiovascular Medicine, The Second Xiangya Hospital，Central South University，Changsha，Hunan 410011，China

*Corresponding author: Xiangping Chai, [chaixiangping@csu.edu.cn](mailto:chaixiangping@csu.edu.cn) and Xinqun Hu, [huxinqun@csu.edu.cn](mailto:huxinqun@csu.edu.cn)

Phone number:8615084714930

**Supplementary table 1：The association between WC and cognitive function during the follow-up period, adjusted for potential confounders**

|  | Model 1 | Model 2 | Model 3 |
| --- | --- | --- | --- |
| **DSST (per SD increase)** | 1.05(0.52, 1.59) ***** | -0.14(-0.59, 0.31) | 0.01(-0.46, 0.48) |
| **MMSE (per SD increase)** | 0.33(0.25, 0.42) ***** | 0.14(0.06, 0.21) ***** | 0.15(0.07, 0.22) ***** |
| **RAVLT (per SD increase)** | 0.03(-0.07, 0.12) | 0.00(-0.08, 0.08) | 0.02(-0.06, 0.11) |
| **STROOP (per SD increase)** | -0.49(-1.00, 0.03) | 0.38(-0.11, 0.87) | 0.22(-0.29, 0.73) |

Model 1: unadjusted

Model 2: adjusted for age, sex, race, glycemic control strategy, education levels, and follow-up period.

Model 3: adjusted for age, sex, race, glycemic control strategy, education levels, and follow-up period, CVD history, heart failure, depression, current smoker, glycosylated hemoglobin, estimated glomerular filtration rate, high density lipoprotein, Low density lipoprotein, systolic blood pressure, diastolic blood pressure.

DSST: Digit Symbol Substitution Test; MMSE: Mini-Mental State Exam; RAVLT: the Rey Auditory Verbal Learning Test;

***** P<0.05

**Stable 2：Association between WC and brain structure area during the follow-up period, adjusted for potential confounders.**

|  | Model 1 | Model 2 | Model 3 |
| --- | --- | --- | --- |
| **TBV (per SD increase)** | 16.46(9.19, 23.74) | 5.08(-1.36, 11.53) | 9.09(2.14, 16.05) * |
| **AWM (per SD increase)** | -0.42(-0.79, -0.05) | -0.24(-0.61, 0.13) | -0.19(-0.57, 0.20) |
| **AGM (per SD increase)** | -0.02(-0.08,0.04) | -0.01(-0.08,0.05) | 0.01(-0.06, 0.07) |
| **ABG (per SD increase)** | -0.04(-0.09, 0.01) | -0.05(-0.10, 0.00) | -0.02(-0.07,0.04) |

Model 1: unadjusted

Model 2: adjusted for age, sex, race, glycemic control strategy, education levels, and follow-up period.

Model 3: adjusted for age, sex, race, glycemic control strategy, education levels, and follow-up period, CVD history, heart failure, depression, current smoker, glycosylated hemoglobin, estimated glomerular filtration rate, high density lipoprotein, Low density lipoprotein, systolic blood pressure, diastolic blood pressure.

TBV: Total brain volume; AWM: Abnormal white matter; AGM: Abnormal grey matter; ABG: Abnormal basal ganglia.

* P<0.05

**Stable 3：Association between BMI and our predefined outcomes during the follow-up period, adjusted for potential confounders**

|  | **Model 3+Medications (Per SD increase of WC)** |
| --- | --- |
| DSST | -0.01(-0.48, 0.47) |
| MMSE | 0.90(0.01,0.17) * |
| RAVLT | 0.10(0.02,0.19) * |
| STROOP | 0.50(-0.01,1.01) |
| TBV | 8.01(0.71, 15.3) * |
| AWM | -0.25(-0.64,0.15) |
| AGM | -0.01(-0.08,0.06) |
| ABG | -0.04(-0.10,0.01) |

Adjusted for Model 3 （age, sex, race, glycemic control strategy, education levels, and follow-up period, CVD history, heart failure, depression, current smoker, glycosylated hemoglobin, estimated glomerular filtration rate, high density lipoprotein, Low density lipoprotein, systolic blood pressure, diastolic blood pressure）in addition to medications(metformin, thiazolidinedione, insulin, statin, sulphonylurea)

DSST: Digit Symbol Substitution Test; MMSE: Mini-Mental State Exam; RAVLT: the Rey Auditory Verbal Learning Test; TBV: Total brain volume; AWM: Abnormal white matter; AGM: Abnormal grey matter; ABG: Abnormal basal ganglia.

* P<0.05

**Supplementary Table 4. Association between brain structure and cognitive function during the follow-up period, adjusted for potential confounders.**

|  | Model 1 | Model 2 | Model 3 |
| --- | --- | --- | --- |
| DSST |  |  |  |
| TBV | 0.04(0.03,0.05) * | 0.02(0.01,0.03) * | 0.02(0.01,0.03) * |
| AWM | -0.5(-0.66, -0.34) * | -0.28(-0.42, -0.13) * | -0.26(-0.41, -0.11) * |
| AGM | -1.94(-2.80, -1.08) * | -1.27(-1.99, -0.55) * | -1.20(-1.93, -0.47) * |
| ABG | -3.73(-5.02, -2.45) * | -2.09(-3.25, -0.94) * | -1.96(-3.12, -0.81) * |
| MMSE |  |  |  |
| TBV | 0.006(0.004,0.008) * | 0.002(0.000,0.004) * | 0.003(0.001,0.004) * |
| AWM | -0.05(-0.08, -0.02) * | -0.02(-0.05,0.00) * | -0.03(-0.06,0.00) * |
| AGM | -0.05(-0.22,0.11) | 0.01(-0.14,0.16) | 0.00(-0.15,0.15) |
| ABG | -0.27(-0.51, -0.04) * | -0.12(-0.34,0.10) | -0.15(-0.37,0.07) |
| RAVLT |  |  |  |
| TBV | -0.002(-0.004,0.000) * | -0.002(-0.004,0.000) * | -0.002(-0.004,0.000) * |
| AWM | -0.06(-0.09, -0.03) * | -0.08(-0.10, -0.05) * | -0.07(-0.10, -0.04) * |
| AGM | -0.09(-0.25, 0.07) | -0.14(-0.29,0.01) | -0.08(-0.23,0.07) |
| ABG | -0.46(-0.69, -0.22) * | -0.56(-0.78, -0.34) * | -0.54(-0.77, -0.32) * |
| STROOP |  |  |  |
| TBV | -0.03(-0.04, -0.02) * | -0.02(-0.04, -0.01) * | -0.024(-0.04, -0.01) * |
| AWM | 0.48(0.29,0.66) * | 0.32(0.12,0.51) * | 0.30(0.10,0.50) * |
| AGM | 0.78(-0.27, 1.82) | 0.56(-0.44,1.56) | 0.42(-0.61,1.46) |
| ABG | 4.15(2.69, 5.62) * | 3.00(1.52,4.66) * | 2.74(1.22,4.26) * |

*Model 1: unadjusted*

*Model 2: adjusted for age, sex, race, glycemic control strategy, education levels, and follow-up period.*

*Model 3: adjusted for age, sex, race, glycemic control strategy, education levels, and follow-up period, CVD history, heart failure, depression, current smoker, glycosylated hemoglobin, estimated glomerular filtration rate, high density lipoprotein, Low density lipoprotein, systolic blood pressure, diastolic blood pressure.*

*DSST: Digit Symbol Substitution Test; MMSE: Mini-Mental State Exam; RAVLT: the Rey Auditory Verbal Learning Test; TBV: Total brain volume; AWM: Abnormal white matter; AGM: Abnormal grey matter; ABG: Abnormal basal ganglia.*

**P<0.05*

**Supplementary Figure 1: Mean cognitive test scores stratified by BMI category at baseline and during the follow-up period**


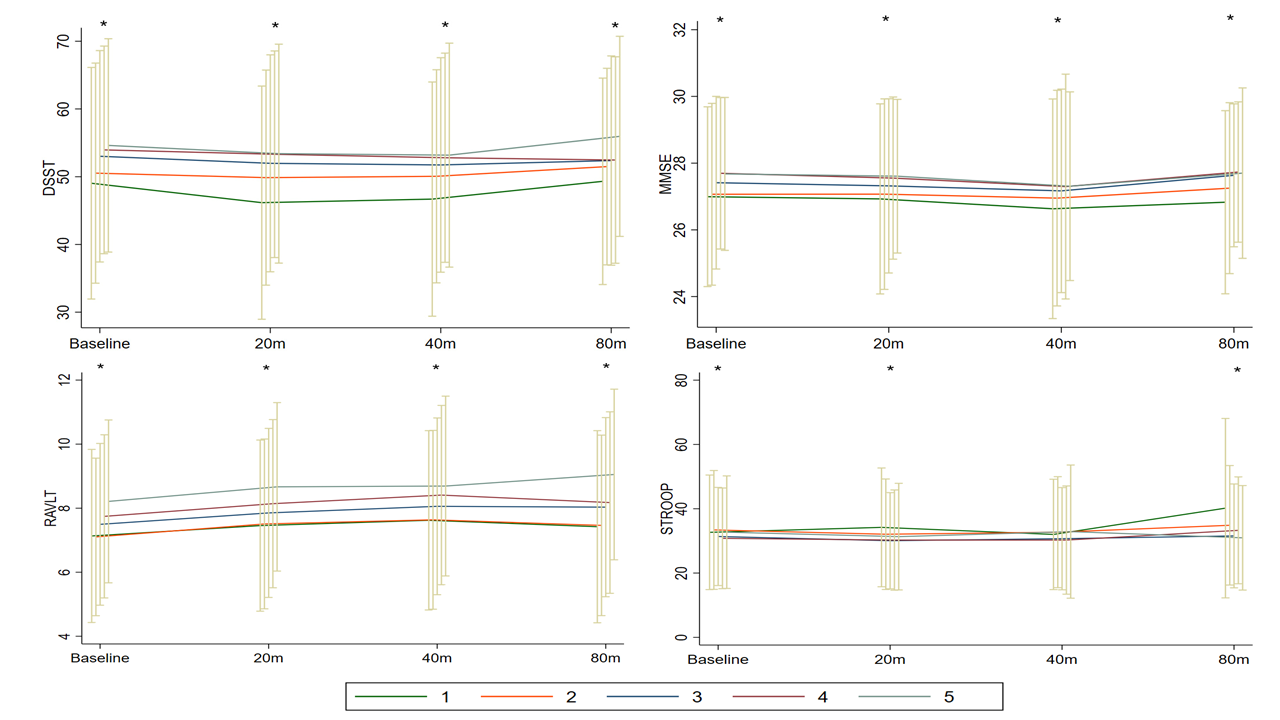


BMI, body mass index; DSST, Digit Symbol Substitution Test; MMSE, Mini-Mental State Exam; RAVLT, Rey Auditory Verbal Learning Test. * P<0.05

**Supplementary Figure 2: Mean area of target brain structure at baseline and during the follow-up period, stratified by BMI category**


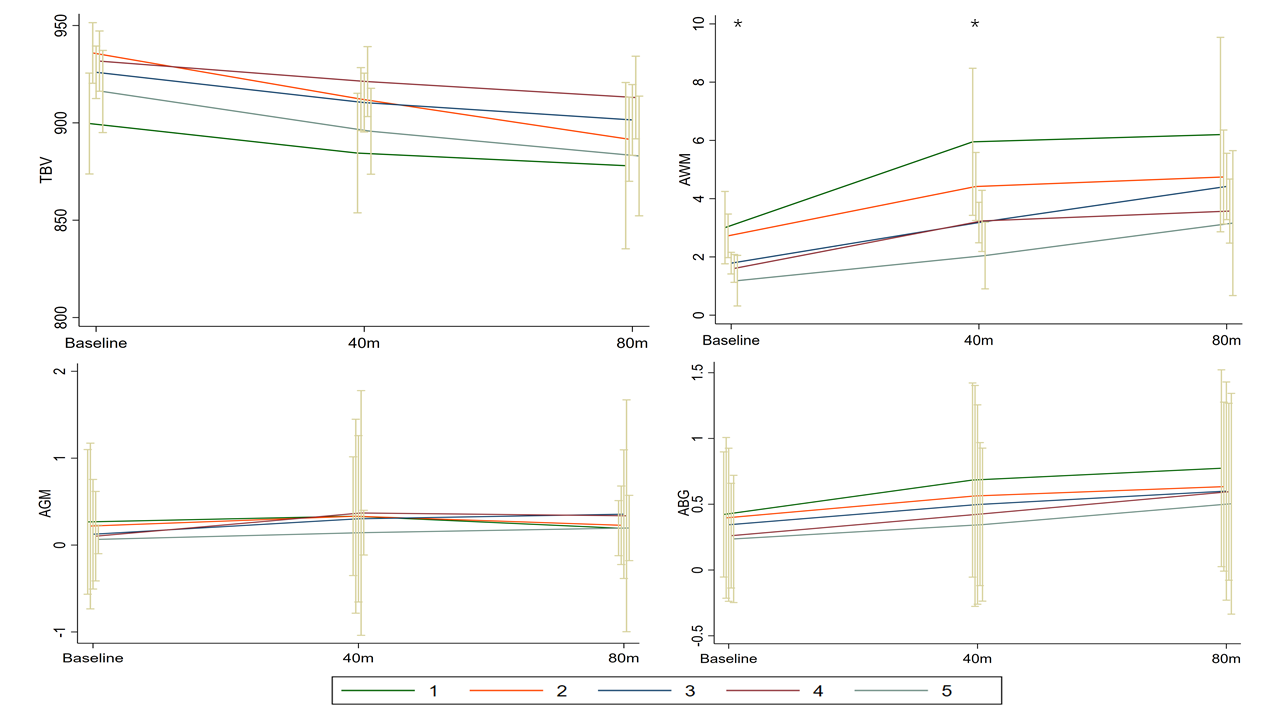


BMI, body mass index; TBV, total brain volume; AWM, abnormal white matter volume; AGM, abnormal gray matter; ABG, abnormal basal ganglia. * P<0.05

**Supplementary Figure 3: Subgroup and interactive analysis of the relationship between BMI and cognitive function during follow-up period.**


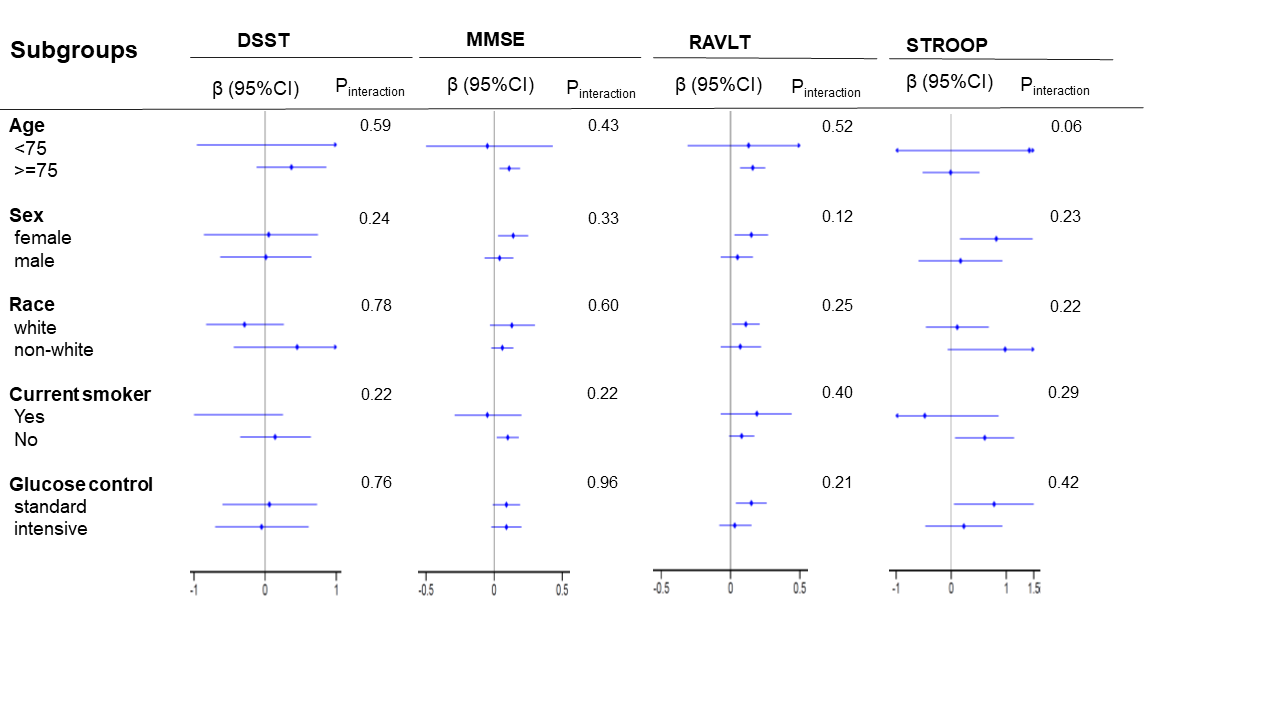


**Supplementary Figure 4: Subgroup and interactive analysis of the relationship between BMI and area of brain structure during follow-up period.**


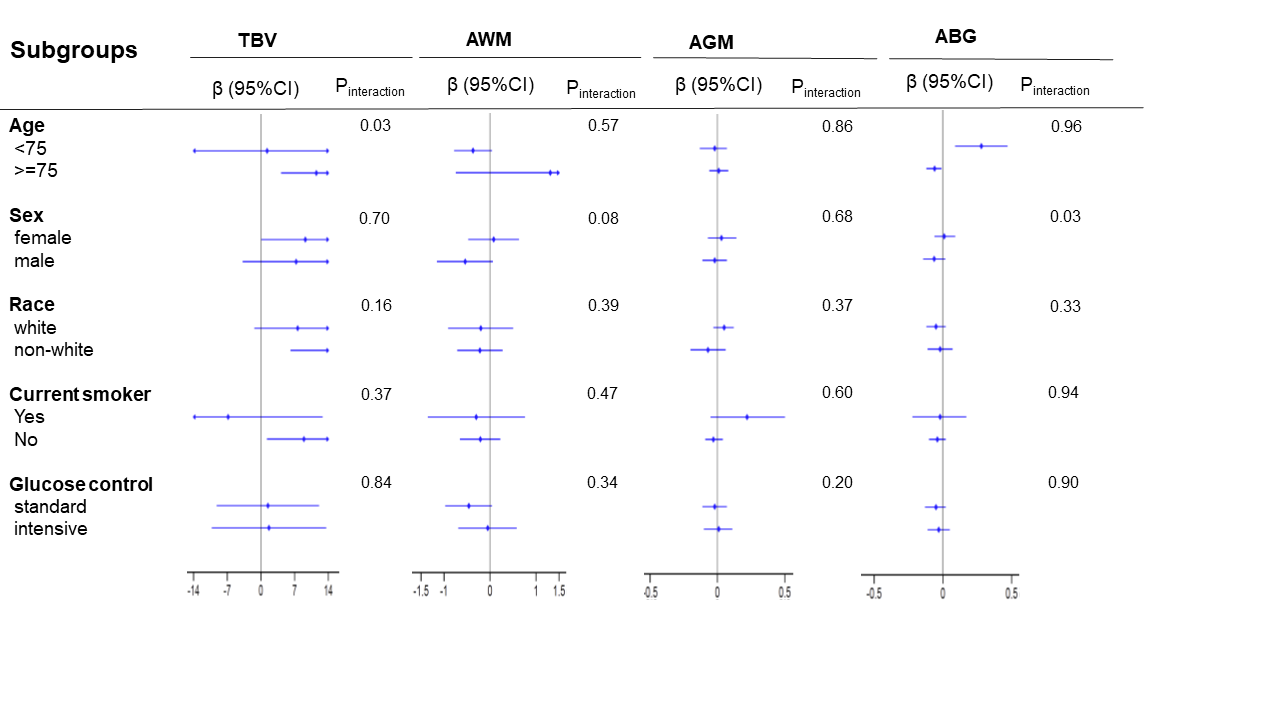

Supplement: Supplementary file 1 [file DataSheet_1.docx]
